# Incarcerated Stomach Within an Inguinal Hernia: A Rare Surgical Presentation

**DOI:** 10.7759/cureus.94873

**Published:** 2025-10-18

**Authors:** Charlotte Kennedy, Ashuvini Mahendran, Md Abu Kamal Nahid

**Affiliations:** 1 General Surgery, Great Western Hospital Academy, Swindon, GBR

**Keywords:** gastric complications, giant inguinoscrotal hernia, incarcerated inguinal hernia, inguinal hernia complications, rare form inguinal hernia, rare hernia, scrotal hernia, stomach herniation, surgery of the stomach, symptomatic hernia

## Abstract

The herniation of the stomach into an inguinoscrotal hernia is exceptionally rare, with very few cases described in surgical literature. The recognition of this presentation is important due to the risk of complications such as obstruction, ischaemia and perforation. We present the case of an elderly gentleman who presented with persistent vomiting and a large, tender left inguinoscrotal hernia. Computed tomography (CT) imaging confirmed the herniation of the stomach into the left inguinal canal with features suggestive of hypoperfusion. The patient underwent an emergency exploratory laparotomy to assess for gastric ischaemia, followed by gastropexy. The patient had an uncomplicated recovery and was discharged with an outpatient follow-up appointment. The awareness of this rare presentation is essential for the timely management and prevention of life-threatening complications.

## Introduction

Inguinal herniae are a common clinical finding, most often containing the omentum or small bowel. In contrast, the herniation of the stomach into the inguinal canal is exceptionally rare, with 21 cases reported in the literature between 1942 and 2020 [1]. These herniae are typically large, longstanding and associated with increased intra-abdominal pressure [2]. Because gastric involvement is unexpected, diagnosis is often delayed until patients present with complications such as obstruction, ischaemia or perforation, all of which carry a significant risk of mortality [3].

We present this case to highlight the importance of the early recognition of gastric herniation within an inguinoscrotal hernia, discuss the associated diagnostic challenges and contribute to the limited existing literature on its surgical management.

## Case presentation

**Figure 1:**
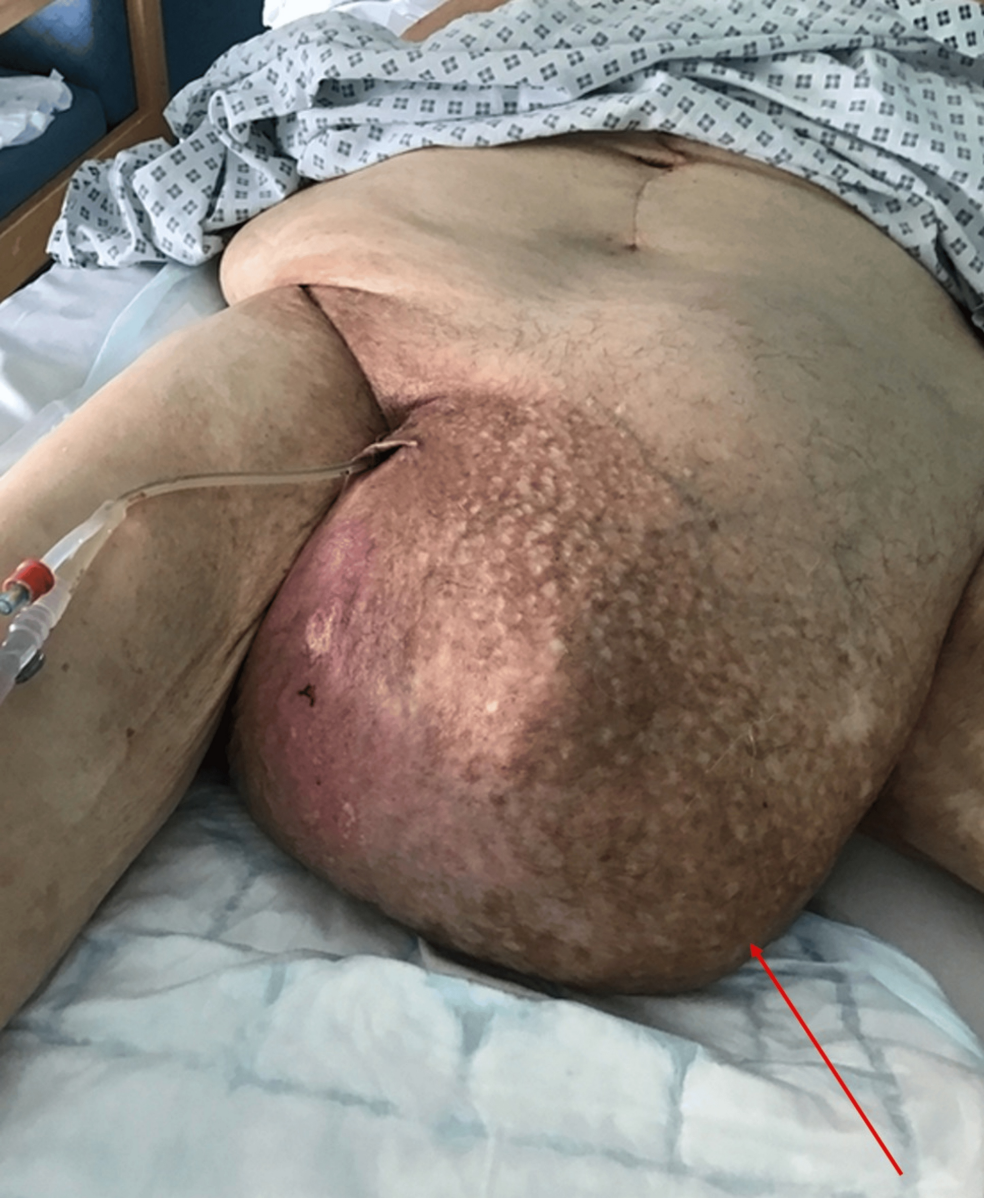
Image of an inguinoscrotal hernia in a patient presenting to the emergency department. The red arrow points to the inguinoscrotal hernia.

An 86-year-old gentleman presented to the emergency department in the United Kingdom with a 36-hour history of nausea, non-bilious vomiting and reduced oral intake. He denied abdominal pain or a change in bowel habits. The patient was a non-smoker with a history of hypertension and a previously recorded body mass index (BMI) of 43. He had previously worked as a farmer, an occupation involving heavy lifting and labour. On examination, a large, soft and tender left inguinoscrotal swelling measuring approximately 35 cm was noted (Figure 1). The manual reduction of the hernia was possible but associated with moderate pain.

Differential diagnoses included inguinoscrotal hernia, groin haematoma or abscess, testicular tumour, hydrocoele, scrotal lymphoedema and other hernia variants.

**Figure 2:**
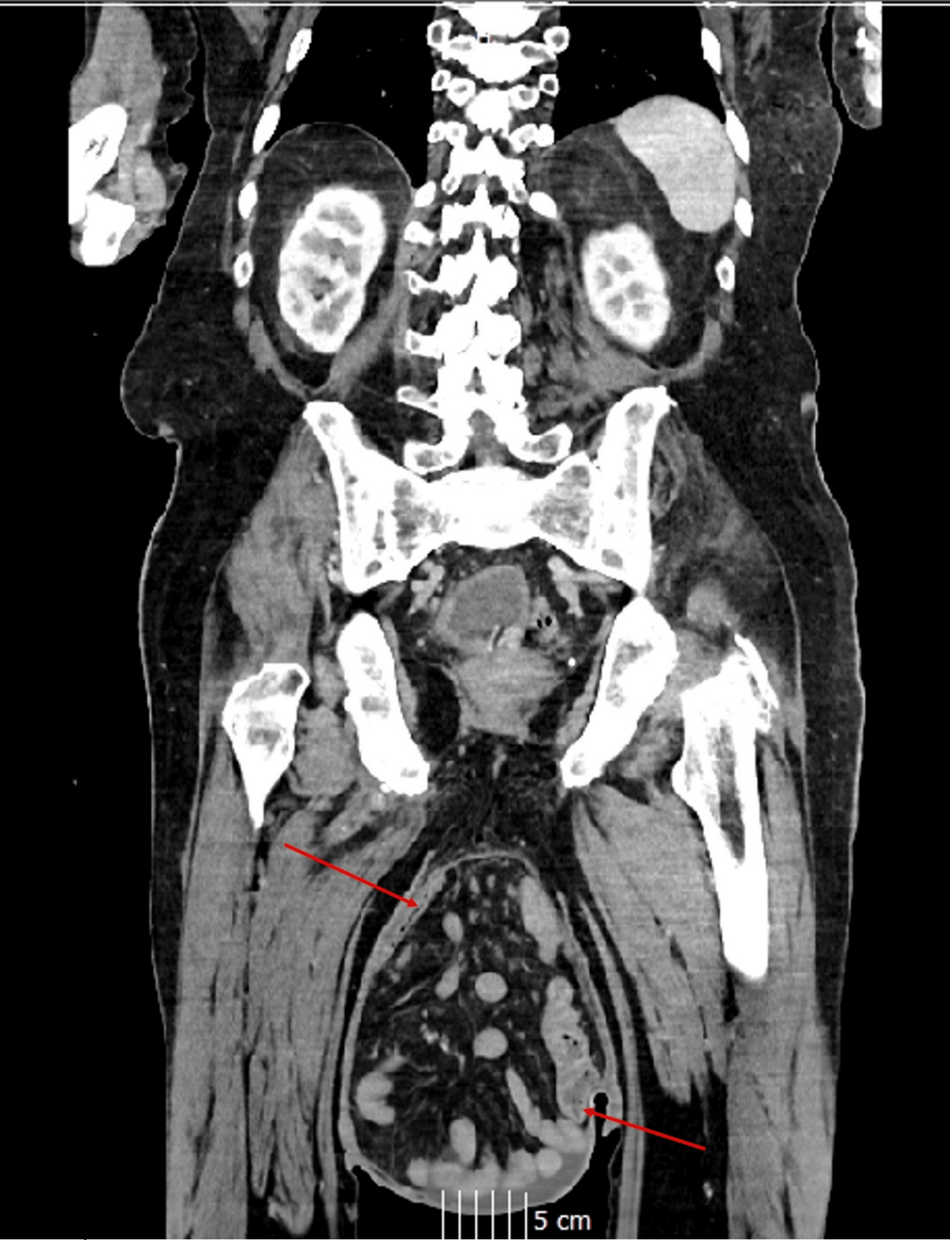
CT imaging of the abdomen and pelvis in a coronal view. Red arrows point to the inguinoscrotal hernial sac containing the stomach and large/small bowel. CT: computed tomography

**Figure 3:**
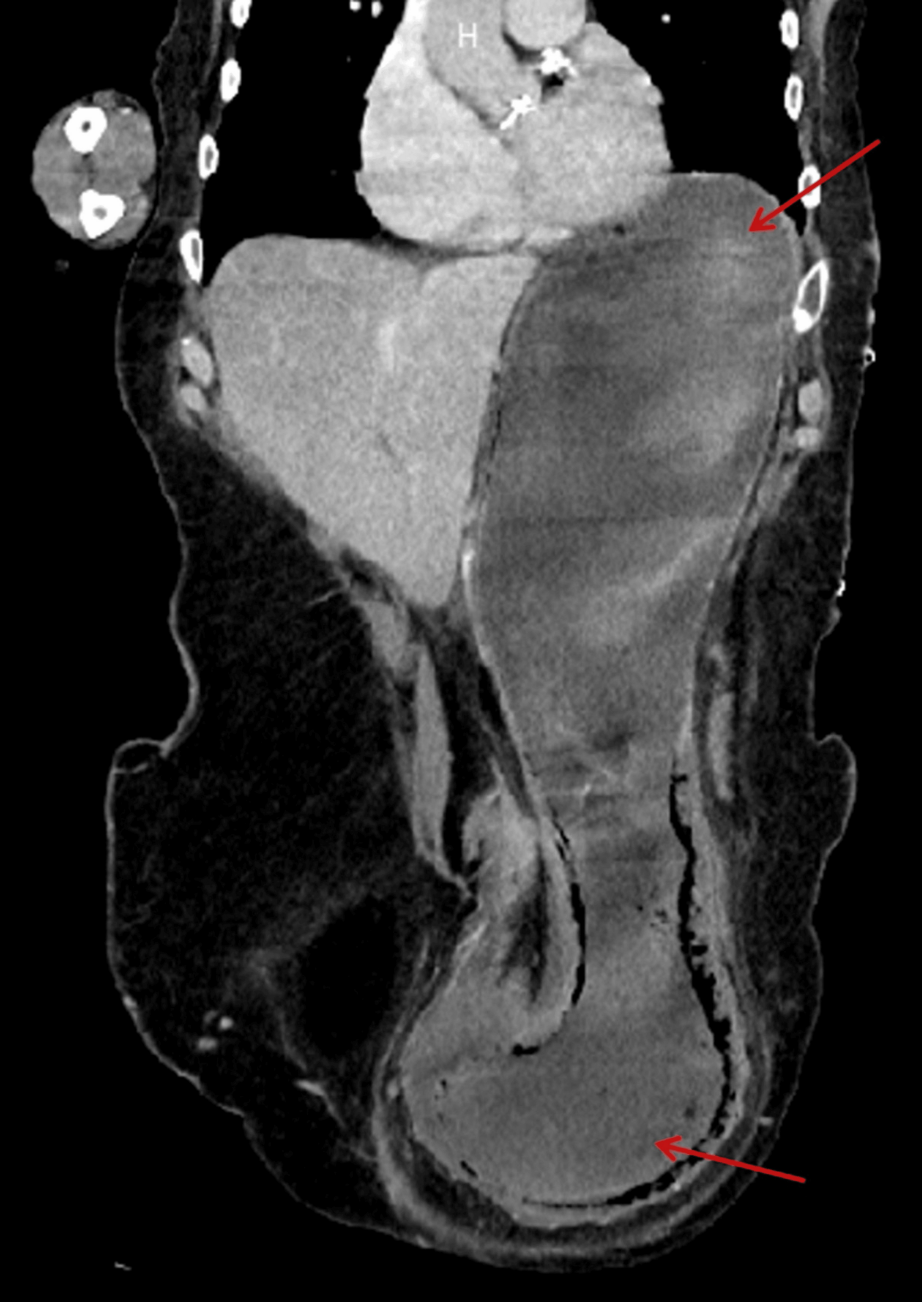
CT imaging of the abdomen and pelvis in a coronal view. Red arrows indicate the descent of the stomach into the inguinoscrotal hernia sac. CT: computed tomography

A computed tomography (CT) of the abdomen and pelvis demonstrated a large indirect left inguinal hernia containing the distal stomach, along with loops of the small and large bowel. The stomach appeared distended within the hernia sac, with surrounding fat stranding suggestive of ischaemia, although no perforation was seen (Figures 2, 3).

Laboratory investigations (Table 1) revealed an elevated lactate level, which was suggestive of tissue hypoperfusion. This raised concerns for gastric ischaemia secondary to strangulation within the hernia sac.

**Table 1:** Laboratory parameters on arrival to the emergency department.

Laboratory Test	Value at Presentation	Reference Ranges
Lactate	2.7	0.6-2.0 mmol/L
Base excess	15.9	-2 to +3 mmol/L
White blood cells	12.71	3.7-11.0 × 10^9^/L
C-reactive protein	38.3	0-5 mg/L
Estimated glomerular filtration rate	86	≥60 mL/minute/1.73 m²

Management and outcome

Management decisions were guided by the patient’s clinical presentation, imaging findings and laboratory results. CT imaging demonstrated a distended stomach with surrounding fat stranding within an inguinal hernia, while blood results were suggestive of gastric outlet obstruction and tissue hypoperfusion. In view of these findings, the patient underwent an emergency laparotomy and remained haemodynamically stable intra-operatively.

During the procedure, the left inguinal hernia sac was found to contain the greater curvature and pyloric antrum of the stomach, loops of the small bowel and a part of the sigmoid colon. All herniated contents were viable and reducible. Given the significant gastric distension observed and the increased risk of gastric volvulus, a gastropexy was performed to anchor and secure the stomach, thereby reducing the risk of future torsion and herniation. Definitive hernia repair was deferred to minimise the risk of intra-abdominal compartment syndrome, with a staged repair using permanent prosthetic mesh planned [4]. Postoperative recovery was uneventful, and the patient was discharged with outpatient follow-up.

## Discussion

The mechanisms underlying the herniation of the stomach into the inguinal canal remain unclear. Anatomically, the stomach is secured by the oesophageal hiatus, gastrocolic ligament and hepatogastric ligament [5]. Inferior attachments, particularly to the greater omentum and gastrocolic ligament, are relatively weak compared to superior fixations [6]. The descent of the greater omentum into a hernia sac may act as an anchor, drawing the stomach downward [7]. Chronic elevations in intra-abdominal pressure, from factors such as chronic cough or heavy lifting, predispose individuals to inguinal hernia formation [8,9]. In this case, the patient’s occupation as a dairy farmer and history of high BMI are risk factors that are likely to have favoured hernia development.

A systematic review has identified 21 cases of stomach-containing inguinoscrotal herniae [1]. Symptoms of uncomplicated herniae included abdominal pain, groin discomfort and a sensation of heaviness or dragging [10]. Of these cases, 94% presented acutely due to gastric outlet obstruction or perforation, requiring surgical management. Strangulated herniae can compromise the blood supply, requiring emergency surgery associated with higher mortality and prolonged recovery compared to planned repair [11]. Early intervention is preferable, as pain, irreducibility and adhesions tend to worsen over time [12,13]. Key principles in management include assessing the reducibility and viability of hernia contents intra-operatively. In large herniae, reduction and herniorrhaphy can predispose to intra-abdominal compartment syndrome, warranting caution during primary repair [14].

Clinical examination frequently provides critical diagnostic clues of strangulation or incarceration [11], with CT being the modality of choice for confirmation and preoperative assessment [15]. Heylen et al. further recognised that CT was used to diagnose stomach-containing inguinoscrotal herniae in 14 cases (67%). The remaining cases were diagnosed before advanced CT scanning techniques, relying on barium swallow studies or clinical and operative findings [1]. In this case, CT imaging provided a thorough assessment of the size and contents of the hernia, as well as potential associated complications.

Management strategies varied across reported cases, with no single technique consistently used [1]. In patients with gastric perforation, all underwent surgery through different approaches, including midline laparotomy with groin incision, laparotomy alone or laparoscopic repair. In cases of gastric outlet obstruction without perforation, both conservative and operative treatments were employed. This variation highlights the absence of a standardised management technique for stomach-containing inguinoscrotal herniae.

## Conclusions

This case highlights a rare presentation of an inguinoscrotal hernia containing the stomach. Prompt recognition and management are critical to avoid life-threatening complications. Where feasible, timely elective surgical repair should be prioritised to minimise the risks associated with emergency intervention. Clinicians should maintain a high index of suspicion for this unusual diagnosis, particularly in patients with large or longstanding herniae. The awareness of this presentation and adherence to image-guided management are crucial to minimise the risk of iatrogenic complications and ensure timely surgical intervention. This is significant because CT imaging serves as a valuable tool for identifying gastric contents within an inguinoscrotal hernia and was essential in determining the contents in this case.

By presenting this case, we contribute to the small body of surgical literature and help expand the understanding of the clinical presentation, diagnostic challenges and management considerations of this rare presentation. The development of provisional guidance and standardised surgical techniques for stomach-containing inguinoscrotal herniae through the reporting and analysis of these cases may help to improve clinical outcomes.
